# Metabolic Acidosis in Leukemia

**DOI:** 10.7759/cureus.17732

**Published:** 2021-09-05

**Authors:** Jaskamal Padda, Khizer Khalid, Varsha Kakani, Ayden Charlene Cooper, Gutteridge Jean-Charles

**Affiliations:** 1 Internal Medicine, JC Medical Center, Orlando, USA; 2 Internal Medicine, Advent Health & Orlando Health Hospital, Orlando, USA

**Keywords:** metabolic acidosis, leukemia, lactic acidosis, warburg effect, atp synthesis

## Abstract

In 2020, the incidence of leukemia was 474,519 with 311,594 mortality worldwide. In 2021, the American Cancer Society (ACS) has estimated 61,090 new cases of leukemia to occur within the United States. It has also been reported that the most common cause of death in children from one to fourteen years old is oncological, with leukemia being the most frequent cause. A phenomenon known as the Warburg effect has been affiliated with cancer. The Warburg effect is a metabolic abnormality of lactic acidosis in malignancies, with most cases presenting as hematological malignancies such as leukemia. Although many theories have been formulated to clarify the role of the Warburg effect, the exact role still remains uncertain. Four suggested theories on why the Warburg effect happens to include cell signaling, adenosine triphosphate (ATP) synthesis, biosynthesis, and the tumor microenvironment. The Warburg effect occurs in leukemia with the help of enzymes such as pyruvate kinases M2 (PKM2), lactate dehydrogenase A (LDHA), pyruvate dehydrogenase kinase 1 (PDK1), and fibroblast growth factor receptor 1 (FGFR1). In this literature, we explain the proposed hypotheses of the Warburg effect, along with the molecular mechanism of how leukemia is able to produce lactic acid, with the intent to better understand this phenomenon.

## Introduction and background

In 2020, 474,519 new cases of leukemia with 311,594 deaths were recorded globally. Out of the 474,519 cases, 269,503 of them were males and 205,016 leukemia cases were in females [1]. Cancer incidence and mortality are estimated within the United States on a yearly basis by the ACS. In 2021, ACS has estimated the incidence of leukemia to be 61,090, with 35,530 cases occurring in males and 25,560 occurring in females [2]. The estimated incidence of acute lymphocytic leukemia (ALL) is 5,690, chronic lymphocytic leukemia (CLL) is 21,250, acute myeloid leukemia (AML) is 20,240, chronic myeloid leukemia (CML) is 9,110 and all other leukemias is 4,800 [2]. The most common cause of death in children aged one to fourteen within the United States is cancer, with leukemia being the most common cancer [2]. Lactic acidosis is a type of metabolic acidosis that can be associated with malignancy, with most being affiliated with hematological malignancies such as leukemia. This phenomenon is referred to as the Warburg effect [3]. Many theories have been formulated to explain the role of the Warburg effect, but the exact purpose still remains a mystery. In this review, we examine these theories to better comprehend the association of the Warburg effect in leukemias.

## Review

Leukemia

**Figure 1:**
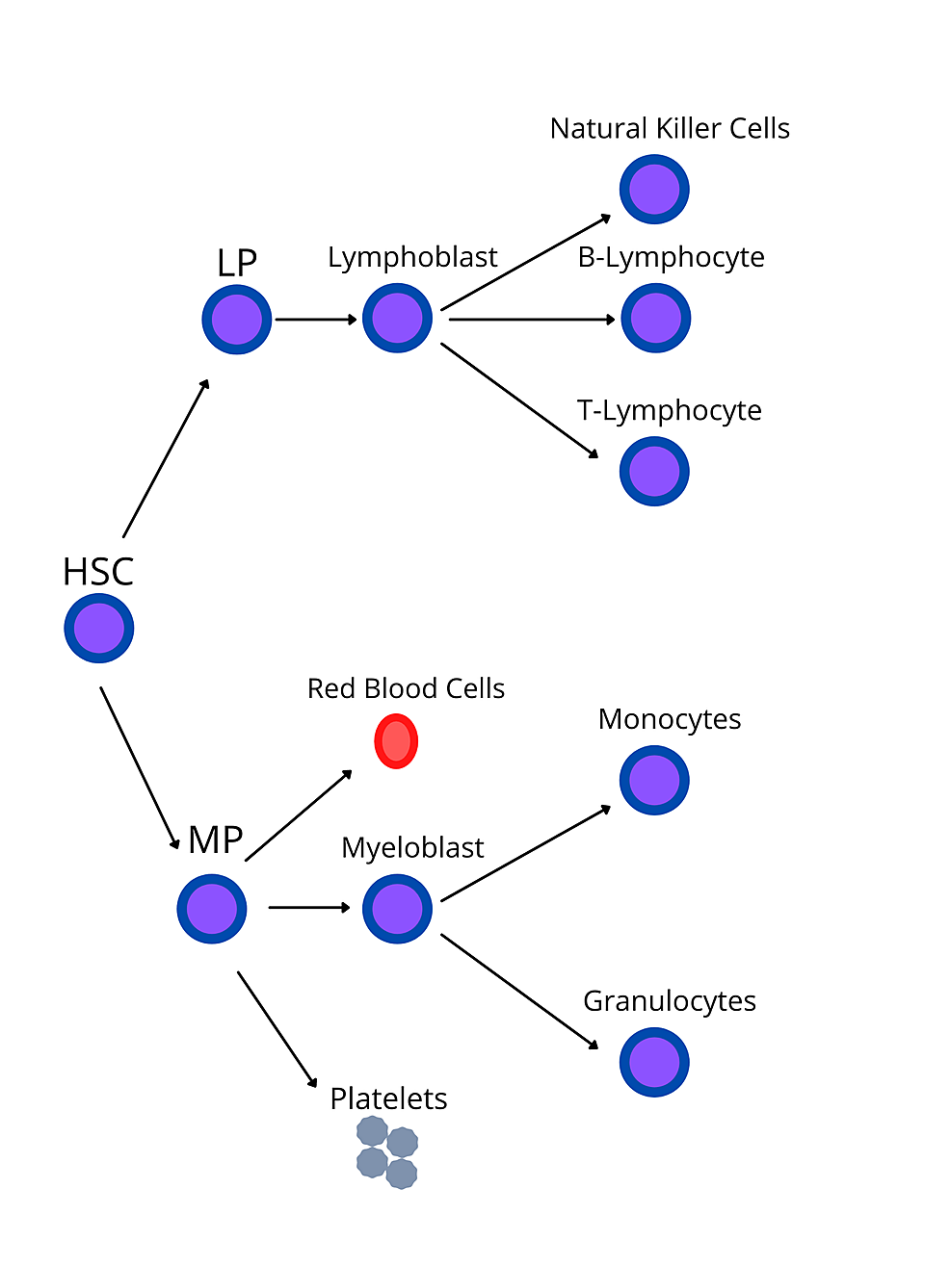
Stem cell differentiation into myeloid and lymphoid lineage HSC – Hematopoietic Stem Cell, LP – Lymphoid Progenitor, MP – Myeloid Progenitor [4-6]. Image created by Jaskamal Padda (author).

Leukemia is a hematological malignancy that originates in the bone marrow [4, 5]. Leukemia normally comprises of white blood cells (WBCs), which are the body’s defense against infections [4]. Growth and division of normal cells generally occur in an orderly fashion when the body requires them, but abnormal cells grow in an uncontrolled manner [4]. This leads to the bone marrow generating an increased quantity of abnormal cells that spill out into the bloodstream [4]. Leukemias can be classified by two methods: the type of blood cells affected and how fast the disease progresses [5]. The progression of the disease can be either acute or chronic. Acute leukemia has a sudden onset and progresses quickly. They are immature blood cells known as blasts that proliferate rapidly. On the other hand, chronic leukemia has slow development over a longer period of time, and these cells are mature [4, 6]. The types of cells that can be involved are either myeloid (red blood cells, platelets, granulocytes, and monocytes) or lymphoid cells (lymphocytes and natural killer cells) (Figure 1). Thus, leukemia can be divided into AML, ALL, chronic myelogenous leukemia, and CLL [4-6]. 

Lactic acidosis 

Lactic acidosis is a metabolic abnormality that has been reported in leukemic patients. It can be divided into type A and type B depending on tissue oxygenation and perfusion. Type A occurs when there is diminished oxygenation to tissues, while type B occurs when there is adequate tissue perfusion. Type B is mainly caused by malignancy, drugs, renal failure, hepatic failure, and diabetes mellitus [3]. Lactic acidosis is caused by a disparity in the production and consumption of lactate. Lactate dehydrogenase is the enzyme that converts pyruvate to lactate under anaerobic conditions. Lactic acidosis has been affiliated with many malignancies, with hematological malignancies being the most common such as leukemias. In malignant cells aerobic glycolysis takes place, which produces lactic acid in the presence of oxygen, this is known as the Warburg effect [3].

Warburg effect 

**Figure 2:**
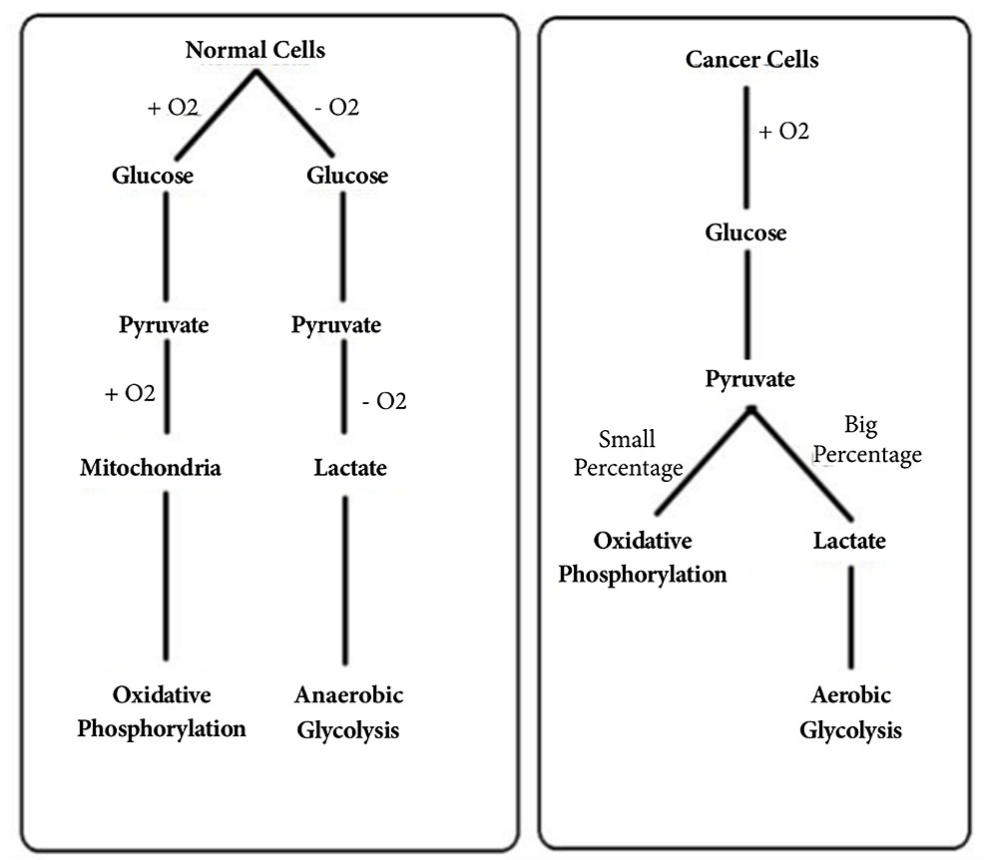
Glucose metabolism: normal cells vs cancer cells. [3]. O2 - oxygen

In 1925, Warburg O noticed that neoplastic cells were taking up and consuming increased quantities of glucose when compared to nonproliferating cells. The glucose was being converted into lactate despite the amount of oxygen available and normal functioning mitochondria. This phenomenon is known as the Warburg effect [7-9]. In normal conditions the Pasteur effect takes place. The Pasteur effect causes inhibition of anaerobic fermentation in the presence of oxygen [10]. In the Pasteur effect, it was also noted that glucose was more consumed anaerobically than aerobically [11]. When oxygen is present, nonproliferating cells consume glucose via the citric cycle and electron transport chain for adenosine triphosphate (ATP) production, while neoplastic cells favor the conversion of pyruvate to lactic acid (Figure 2) [3]. It is yet unknown why neoplastic cells utilize a less efficient method to acquire ATP. It is hypothesized that the purpose is beyond ATP production [9]. Four functions have been proposed to hypothesize the purpose of the Warburg effect: cell signaling, rapid ATP synthesis, biosynthesis, and tumor microenvironment [8].

Cell signaling 

The Warburg effect has been associated with direct signaling functions in regard to tumor cells. This association with signal transduction is able to affect other cellular processes which promote tumorigenesis. The Warburg effect is connected to two areas of direct signaling links which are the modulation and generation of reactive oxygen species (ROS) and the modulation of chromatin state [8]. It is important to have an appropriate balance of ROS. Excessive ROS has many harmful effects including damage to the cell membrane, nucleic acids, and much more, whereas low levels of ROS disrupt signaling processes that are needed for cell proliferation. ROS has both antitumorigenic and pro-tumorigenic effects. The Warburg effect essentially modifies the mitochondrial redox potential which results in changes in ROS production [8, 12]. Greater amounts of superoxide and hydrogen peroxidase are produced in cancer cells compared to normal cells. Cancer cells also have greater amounts of antioxidants compared to normal cells to improve their survival in pro-oxidizing situations, by neutralizing ROS. ROS demonstrates a critical role in cell signaling leading to tumor metastasis, proliferation, and cell survival [12]. ROS inactivates phosphatase and tensin homolog (PTEN) along with activating the phosphoinositide 3-kinase/protein kinase B/mammalian target of the rapamycin (PI3K/AKT/mTOR) pathway and hypoxia-inducible factor (HIF) [12, 13]. PTEN is needed for the initiation of apoptosis. ROS oxidizes PTEN leading to loss of its function and subsequent activation of PI3K/AKT/mTOR [14]. PI3K/AKT/mTOR activates RAS-mitogen activated protein kinase (RAS-MAPK) and phosphoinositide 3-kinase/protein kinase B/endothelial nitric oxide synthase (PI3K-AKT-eNOS) pathways [14]. Cell proliferation is obtained by RAS-MAPK while PI3K-AKT-eNOS leads to cell survival [12, 14]. Activation of HIF allows cancer cells to survive in hypoxic conditions allowing them to adapt to the environment to further permit survival and growth [14]. Modulation of chromatin through a signaling link between the metabolism of glucose is equally important [8]. Metabolism of glucose by aerobic glycolysis in cancer cells influences chromatin by histone acetylation. The responsibility of chromatin is to regulate deoxyribonucleic acid (DNA) repair, gene transcription, and cancer progression [15]. One crucial way neoplastic cells obtain resistance to DNA damaging drugs is by increasing DNA repair activity [15]. It is recognized that the substrate for histone acetylation, acetyl-coenzyme (CoA), can be regulated by glucose flux [8]. Liu et al. reported that glycolysis inhibition with genetic approach or medications caused histone hypoacetylation leading to compact chromatin. This threatened the efficacy of DNA repair and allowed for DNA damaging drugs to be more sensitive in cancer cells. On the other hand, reversal of compact chromatin was noted when histone acetylation was restored [15]. Acetyl-CoA is a product of the glycolysis pathway that delivers the acetyl group which is needed for histone acetylation. Studies have revealed that intracellular acetyl-CoA levels are associated with the promotion of a direct connection between cellular metabolism and the regulation of growth genes [8, 15]. ATP-citrate lyase (converts citrate to acetyl-CoA) is able to push cells into the growth phase through its effect on histone acetylation. On the other hand, loss of histone acetylation is seen with decreased glucose or ATP-citrate lyase levels. Therefore, there is a connection between glucose metabolism and histone acetylation which impacts chromatin structure. Ultimately, acetylation is induced by the availability of nutrients which proves that its status is the result of the Warburg effect [8].

ATP synthesis

Aerobic glycolysis is the production of two ATP molecules from the metabolism of one glucose molecule to lactate, while oxidative phosphorylation utilizes one glucose molecule to produce 36 ATP molecules [16]. Which raises the question, why do neoplastic cells run an inadequate method to produce ATP? Aerobic glycolysis metabolizes glucose up to 100 times faster than oxidative phosphorylation. Hypothetically, the rate of ATP production may be greater in cells using aerobic glycolysis when competing for limited and shared energy resources [8]. A study by Epstein et al. showed that when alterations were made to the cell membrane ATP-dependent pumps to increase cellular ATP demand, there was a rapid increase in aerobic glycolysis, but oxidative phosphorylation remained constant [17].

Biosynthesis

The Warburg effect has been postulated to be a mechanism to help in the synthesis of biosynthetic requirements in neoplastic cells. As glucose is metabolized, carbon molecules are accumulated which can further be used for anabolic processes to maintain cellular proliferation. These carbon molecules are used in the generation of proteins, lipids, and nucleotides which can further be redirected into different pathways that branch off of glycolysis [8, 13]. Another theory is that neoplastic cells require more reducing equivalents, such as nicotinamide adenine dinucleotide phosphate, that favor tumorigenesis and promote anabolism by increasing antioxidant defense to protect it from radiation, chemotherapy, and aggressive environments. There is increased production of these reducing equivalents in the hexose monophosphate shunt as there is increased glucose consumption [13]. It has also been suggested that the Warburg effect is required for the regeneration of oxidized nicotinamide adenine dinucleotide from reduced nicotinamide adenine dinucleotide (NADH) in aerobic glycolysis during the conversion of pyruvate to lactate. This is believed to occur to keep glycolysis going during the glyceraldehyde phosphate dehydrogenases step where NADH is produced. It is assumed that the Warburg effect encourages rapid biosynthesis which in turn supports proliferation and growth [8].

Tumor microenvironment

The Warburg effect causes acidification of the tumor microenvironment which presents as an advantage to tumor cell growth and metastasis. Increased glucose metabolism causes an increased production of lactate, resulting in an acidic microenvironment [18]. It is suggested that the increased acidic environment allows for more enhanced growth, invasiveness, and metastasis of tumor cells [8, 19]. The theorized mechanism by which these events occur is by increasing lactic acid production via aerobic glycolysis, causing hydrogen to disperse from the tumor microenvironment into the neighboring normal tissues. This leads to the remodeling of cells and therefore permitting local tissue invasion [18, 19]. In a study conducted by Estrella et al., the potential of hydrogen (pH) of the tumor microenvironment and tumor invasion was monitored over time using intravital microscopy. In every case, the microenvironment which had the lowest pH had the highest tumor invasion, whereas microenvironments with normal or near-normal pH did not have any tumor invasion. Oral administration of sodium bicarbonate was enough to increase the pH in the tumor environment and halt tumor growth and invasion which supports the fact that acidic environments promote tumor metastasis [19]. It was also noted that cells along the invasive edges were positive for sodium-hydrogen exchanger (NHE-1) and the glucose transporter (GLUT-1) by immunohistochemical analysis, which were recognized to be associated with an acidic tumor microenvironment. GLUT-1 is an enzyme that allows the entry of glucose into cells, which is why it is important in facilitating the Warburg effect. Cancer metastasis and invasion have been correlated with abnormal expression of GLUT-1 [19, 20]. Tumor cells maintain the acidic environment in two main mechanisms. Firstly, tumors were shown to develop poorly organized and leaky vasculature which decreases oxygen delivery. The decrease in oxygenation increased glycolytic flux through the Pasteur effect where glucose is consumed more anaerobically, while the Warburg effect is capable of increasing acid production with sufficient oxygen supply [19]. Secondly, poor vasculature perfusion makes it difficult to remove the acid from the environment. This accumulation of acid will result in acid distribution throughout the surrounding tissues. This suggests that tumor cells are able to create an acidic environment that affects normal cells negatively but is non-toxic to malignant cells and promotes local tumor invasion [19]. Though there are many theories why the Warburg effect increases lactate in neoplastic cells, further research is required to determine if the postulated functions account for this phenomenon.

Pyruvate kinases M2 (PKM2), pyruvate dehydrogenase kinase 1 (PDK1), lactate dehydrogenase A (LDHA), and fibroblast growth factor receptor 1 (FGFR1)

In leukemia, PKM2, LDHA, PDK1, and FGFR1 play an important role in establishing the Warburg effect [9, 21-23]. Pyruvate kinase is a rate-limiting enzyme in aerobic glycolysis, that converts phosphoenolpyruvate to pyruvate [9, 21]. There are two subtypes, PKM1 and PKM2. Expression of PKM1 is within tissues utilizing oxidative phosphorylation which requires high levels of ATP, while PKM2 is expressed in neoplastic and embryonic cells [9]. The Warburg effect in leukemia has been attributed to specific enzymes such as PKM2, LDHA, and PDK1. FGFR1 is a tyrosine kinase that can control the activation of PMK2, LDHA, and PDK1 by tyrosine phosphorylation [22]. Stabilization or replacement of PKM2 with PKM1 showed reversal of Warburg effect along with suppression of tumor progression. LDHA is a mediator of aerobic glycolysis which is more potent than PKM2. LDHA inhibition also leads to the inhibition of neoplastic growth. Deletion of LDHA and PKM2 has been shown to cause significant delays in the initiation of leukemia (AML and CML) [9]. PDK1 functions to inactivate pyruvate dehydrogenase (PDH), overall inhibiting pyruvate dehydrogenase complex (PDC) within the mitochondria. The normal role of PDC is to convert pyruvate into acetyl-CoA. This allows for oxidative phosphorylation to occur for ATP production. FGFR1 tyrosine phosphorylation activates PDK1 which inhibits PDH and causes a metabolic switch to aerobic glycolysis from oxidative phosphorylation [23]. A study done by Bonnet showed that inhibition of pyruvate dehydrogenase kinase can revert aerobic glycolysis back to oxidative phosphorylation, further inhibiting tumor growth and decreasing lactic acid levels [24]. 

Lactic acidosis has been reported in many leukemia cases (Warburg effect), some of which are mentioned below [3, 25-26].

Case one

Ghrewati et al. presented a case of a 62-year-old female with a past medical history (PMH) of anemia. She complained of weakness and dizziness for one week and 20lb weight loss over one year. Vitals were within normal limits except blood pressure of 169/72 mmHg and pulse of 102 bpm. Initial lab reports showed a mixed anion gap and non-anion gap metabolic acidosis along with a high lactic acid level. Based on the calculated urine osmolar gap and urine pH, renal involvement suggested RTA. Treatment was initially started with fluids and sodium bicarbonate. Additionally, a bone marrow biopsy was done, which made the diagnosis of B-cell ALL. CT showed bilateral nephromegaly. Though initial treatments with fluids and bicarbonate improved the lactic acid levels, complete resolution was achieved with chemotherapy [3].

Case two

Gardner et al. presented a case of a 77-year-old Caucasian male who was brought to the emergency department after he collapsed. He had malaise, anorexia, weight loss, and epigastric pain after meals for the past six weeks. The initial arterial blood gas analysis showed a high lactate level of 18 mmol/L, along with low bicarbonate of 11 mmol/L, and a pH of 7.27. No liver or renal dysfunction was noted in labs. The diagnosis of lactic acidosis was made. Blood film revealed a myelodysplastic syndrome with abnormally high WBCs. Bone marrow biopsy demonstrated CML. Emergency chemotherapy was initiated and lactic acid levels came down to 4 mmol/L in the next day [25].

Case three

Udayakumar et al. presented a case of a 20-year-old male who was brought to the emergency department with complaints of fever, altered sensorium, and tachypnea. Further investigation revealed a Glasgow coma scale of 6/15, blood glucose of 45mg/dl, serum lactate of 8.2 mmol/L along with high uric acid and low sodium levels. Arterial blood gas analysis showed a pH of 7.05, partial pressure of carbon dioxide (PCO2) of 9.3 mmol, bicarbonate of 9.3 mmol, and an anion gap of 40 mmol. Peripheral blood smear demonstrated myeloblasts and a low platelet count leading to the diagnosis of AML. As the patient developed sudden respiratory arrest, he was intubated. Treatment with hypertonic saline for hyponatremia and bicarbonate therapy for lactic acidosis was initiated along with leukapheresis and chemotherapy (cytarabine and daunorubicin). The patient's acidosis did not improve, and the patient succumbed to illness the next day [26].

Diagnosis

Patients can present with metabolic acidosis at various stages of leukemia [27]. Metabolic acidosis is defined as a decrease in serum bicarbonate concentration less than 21 mEq/L and pH less than 7.35. There is a secondary decrease in arterial partial pressure of carbon dioxide of 1 mmHg for every 1 mmHg fall in serum bicarbonate concentration [28]. Serum electrolytes, arterial blood gas analysis, and serum lactate levels are used to establish the diagnosis of metabolic acidosis. [27-29]. In some cases, acidosis can be the initial presentation of leukemia. Patients presenting with lactic acidosis without clinical evidence of tissue hypoperfusion should raise the question of an underlying malignancy, hematological disorders, or leukemia relapse. This should prompt further testing to establish the diagnosis of which type of leukemia it is with complete blood count and bone marrow biopsy [27]. The increase in lactic acid levels in leukemic patients results from overproduction of lactate from glucose, leading to hypoglycemia in these patients. Blood glucose levels should also be checked in leukemic patients [30]. Friedenberg et al. stated that thiamine deficiency also plays an essential role in the development of lactic acidosis in malignancy. Accordingly, testing for thiamine deficiency should also be included in the diagnostic workup, as it aids in the treatment [31]. Leukemic cells are able to infiltrate the liver and kidneys leading to their enlargement. Clearance of lactate from the body is primarily by the liver and to a lesser degree by the kidneys. Infiltration of these organs leads to decreased lactate clearance further inducing lactic acidosis. Biopsies of the liver and kidneys can help in establishing this type of parenchymal infiltration [27, 30].

Treatment

The main treatment strategies for metabolic acidosis in a leukemic patient include IV fluids, alkalinization with bicarbonate infusion, chemotherapy, dialysis, IV thiamine, and supportive care [27, 29]. Dialysis is recommended in severe cases of metabolic acidosis. Dialysis can be either continuous venovenous hemofiltration (CVVH) or sustained low-efficiency dialysis (SLED). Bicarbonate infusion alone has severe deleterious outcomes such as inconsistent lactic acid production intracellularly and decreased cardiac contractility. This is why it is suggested only in severe metabolic acidosis cases. On the other hand, bicarbonate infusion along with CVVH showed improvement in arterial blood gas values in some patients. Depending on the type of leukemia, chemotherapy with antineoplastic agents has demonstrated superior effects compared to other treatment options for the correction of acidosis in leukemic patients. Even though patients are started with different treatments initially, improvement in arterial blood gas analysis, serum electrolytes, and lactate levels are rapidly seen after the chemotherapeutic agents are started [27, 29]. The use of thiamine is theorized to drive pyruvate into Acetyl CoA rather than lactic acid. Excess thiamine is also dangerous as it is a cofactor for transketolase. It is a critical enzyme in the pentose phosphate pathway that leads to the production of ribose that is required for DNA synthesis which enhances tumor growth. Further research is required for the use of thiamine replacement therapy in the treatment of metabolic acidosis in leukemia. [27, 29].

## Conclusions

Type B lactic acidosis is a form of metabolic acidosis which is a complication in leukemia. The Warburg effect was discovered in 1925 by Warburg O, who observed an increase in lactic acid production in neoplastic cells, with most occurring in hematological malignancies such as leukemia. These cells preferred aerobic glycolysis over oxidative phosphorylation in the presence of oxygen. Why malignancies such as leukemia run an insufficient method to obtain ATP is still unknown, but it is theorized that the Warburg effect transpires for ATP synthesis, biosynthesis, tumor microenvironment, and cell signaling. The mechanism by which leukemic cells increase lactic acid products is via PKM2, PDK1, LDHA, and FGFR1. Inhibition of LDHA, along with stabilization or replacement of PKM2 with PKM1, has demonstrated reversal of the Warburg effect and inhibition of neoplastic growth. The presence of lactic acidosis when there is sufficient oxygen perfusion should raise concerns for malignancies such as leukemia or leukemic relapse since lactic acidosis can be the initial presentation in leukemia. Thiamine deficiency has also been associated with an increased risk of developing metabolic acidosis. The initial treatment modalities for lactic acidosis in leukemic patients include intravenous fluids, sodium bicarbonate, and thiamine replacement. However arterial blood gas, serum electrolytes, and lactate levels improve after the initiation of chemotherapy. Further research is still needed to identify the exact role of metabolic acidosis in leukemia (Warburg effect). This will help in the development of better and advanced treatment options to aid in mortality reduction.
